# A conversation with Keith McCrae, MD, staff and professor of molecular medicine, Cleveland Clinic

**DOI:** 10.1017/cts.2025.10146

**Published:** 2025-09-16

**Affiliations:** Clinical Research Forum, Washington, DC, USA

## Top 10 clinical research achievement awards Q & A

This article is part of a series of interviews with recipients of Clinical Research Forum’s Top 10 Clinical Research Achievement Awards. This interview is with Keith McCrae, MD, Staff and Professor of Molecular Medicine, Cleveland Clinic. Dr McCrae’s research focuses on translational research in vascular biology. He received a 2025 Clinical Research Achievement Award for “Pomalidomide for Bleeding in Patients with Hereditary Hemorrhagic Telangiectasia.” *The interview has been edited for length and clarity.*

## How did you first get interested in clinical research?

There was not a single event. As clinicians, we sometimes see patients and do not understand their underlying disease as well as we would like to, and unfortunately, we do not always have an effective treatment for everything we see. For me, those instances bring out a desire to help the patients, along with a natural curiosity to solve the problem. Ultimately, that leads to clinical research. Through clinical research, we can find solutions that help not only that single patient but hundreds or thousands more. It’s the challenge of answering questions and the opportunities to make a difference in patients’ lives that attracted me to this area of research.

## The award-winning research evaluated the safety and efficacy of pomalidomide for hereditary hemorrhagic telangiectasia (HHT). What inspired this clinical trial?

I first conceived this study 13 – 14 years ago, and it was actually based on one patient. This patient had both HHT and another underlying bleeding disorder, and he was bleeding extensively from his gut and nose, requiring up to four blood transfusions and multiple treatments with coagulation factor concentrates every week. Clearly, that was not sustainable, and it had been recommended that he have his entire small bowel resected. That would have been completely life-changing, of course, so I went to the literature to see if there was any other possible answer. I found a description of a similar patient who had been treated with the drug thalidomide. We tried that, and remarkably, within two weeks, there was a dramatic change. The patient’s bleeding almost entirely stopped, and no more transfusions were needed. Over the next couple months, he was able to resume a more normal life. It was amazing, but we didn’t know if it was a one-off or if this type of treatment could also help others. So, I resolved to study it.

## What happened next?

Clinical research isn’t always a straightforward course, and what came next was several years of trying to find funding and the time to write the study. I eventually got support from Celgene (now Bristol Myers Squibb) for a small pilot study with pomalidomide, a thalidomide analogue, which showed signs of efficacy, and I also got support from NIH Planning Grant Program (R34), which enabled me to write the large trial. Once that was accepted and funded, we assembled our clinical trial sites across the country, got all the contracts signed, and finally launched – just before the onset of the COVID pandemic. Fortunately, we were able to adapt to become mostly virtual, with only one in-person visit at the front and one in-person visit at the end.

## Both the pilot and large trial used the drug pomalidomide, not thalidomide?

Yes. Pomalidomide is a thalidomide derivative; however, it has a different side effect profile, with less neuropathy. It has anti-blood vessel activity, is generally well tolerated, and is used in cancer therapies, primarily to treat multiple myeloma.

## What did the results show?

We found that pomalidomide resulted in a significant, clinically relevant reduction in epistaxis severity and improved disease-specific health-related quality of life for patients with HHT. We had an NIH-designated independent data safety monitoring board that we met with periodically, and ultimately, they recommended stopping the study early because of success.

## Isn’t it rare for a trial to be terminated early because of success?

Yes. The results were quite gratifying to me, to all the investigators, and to everyone involved in trial, including those at NIH and in the patient advocacy group, Cure HHT. Cure HHT was an essential partner for us, helping us dramatically with patient recruitment and education, and then communicating results.

## Is this changing how HHT is treated?

HHT affects about 1 in 3,800 people, and no treatments, including this one, are approved by the U.S. Food and Drug Administration (FDA) or European Medicines Agency. Clearly, though, our result is finding its way into the literature, and I’m hopeful that this research will allow clinicians to lobby more effectively to third-party payers for coverage of this medication as a treatment option. This trial also stimulated interest in HHT, because we showed that studying this disease was feasible and that patients respond to treatment. Now there are a few different pharmaceutical companies developing or running trials of other drugs. As a clinician, you want to have options, and as different treatments emerge, it may be possible to pick and choose which may be most successful approach for each individual patient.

## Where is your research headed next?

We’ve submitted a paper with the results of longer-term follow-up on patients who remained on pomalidomide after the study ended. We are also applying to NIH for funding to characterize blood samples we collected during the study before and after treatment from both placebo-treated patients and pomalidomide-treated patients. That’s never been done, and it might offer additional insights into the biological pathways involved in HHT and other approaches to treating it.

## What advice do you have for someone starting their career in clinical research?

Years ago, I was speaking with a wonderful physician-scientist, Evan Sadler, a former president of the American Society of Hematology. I told him about our results with thalidomide in our patient with HHT, and I remember him saying, “Never ignore an N of 1.” I think that’s great advice. Nowadays, researchers use big data, a tremendous amount of data, and while that’s certainly valuable, I sometimes wonder, if in all those studies with tens of thousands of patients, when we look at effects across such large populations, whether we might be missing some unique cases that we could be learning from. Obviously, we can’t always look at patient-level data. But every clinician is going to come across rare patients who stand out. When you see something like that, do not just accept it. Dive into it. It’s telling you something important. Also, be persistent – with the research and with the grant process. Work with your NIH program officers to help you navigate the system. They will guide you through the different types of grants, ensure you apply to the right program, and keep you moving forward.

